# Dynamic migration of free amino acids from garlic in light soy sauce and its impact on taste formation

**DOI:** 10.3389/fnut.2026.1768181

**Published:** 2026-04-29

**Authors:** Ming-Lu Lan, Lin Wang, Lian He, Hua-Chang Wu, Yu-Wen Yi, Xue-Mei Cai

**Affiliations:** 1 Key Laboratory of Food Nutrition and Health of Sichuan Provincial Universities, Sichuan Tourism University, Chengdu, Sichuan, China; 2 College of Culinary and Food Science Engineering, Sichuan Tourism University, Chengdu, Sichuan, China; 3 Culinary Science Key Laboratory of Sichuan Province, Sichuan Tourism University, Chengdu, Sichuan, China

**Keywords:** electronic tongue, free amino acids, garlic, light soy sauce, multivariate analysis, taste modulation

## Abstract

**Introduction:**

Garlic-flavored light soy sauce is a representative composite seasoning whose taste formation depends not only on fermentation-derived components in light soy sauce but also on the time-dependent transfer of garlic-derived taste substances. However, evidence regarding the dynamic migration of non-volatile taste-active compounds—particularly free amino acids (FAAs)—and their roles in taste formation during soaking remains limited. This study aimed to elucidate the time-dependent migration of garlic-derived FAAs during soaking and their contribution to taste formation, and to assess a practical soaking endpoint.

**Methods:**

Samples at different soaking times were evaluated using electronic tongue analysis and targeted FAA quantification. The soaking procedure was used to establish a controlled experimental model rather than to represent an industrial standard protocol. Taste activity values (TAVs) and orthogonal partial least squares–discriminant analysis (OPLS-DA) with variable importance in projection (VIP) values were used to identify key taste-active and discriminating amino acids and to assess a practical soaking endpoint.

**Results:**

Electronic tongue analysis showed that sourness and bitterness gradually decreased, whereas sweetness and umami exhibited an overall increasing trend with soaking time; the 30- and 40-day samples exhibited highly similar global taste profiles, indicating stabilization of the taste profile. A total of 16 FAAs were detected: total FAAs reached a maximum at day 20, whereas glutamic acid accumulated continuously and reached its highest level at day 30. TAV analysis indicated multiple taste-active amino acids, with glutamic acid making the greatest contribution, and OPLS-DA (VIP values) likewise highlighted glutamic acid among the discriminating variables. These taste shifts were consistent with the time-dependent migration and accumulation of garlic-derived taste-active FAAs, particularly glutamic acid.

**Conclusion:**

Under the tested conditions, glutamic acid is the key driver of umami enhancement and taste stabilization in the garlic–light soy sauce soaking system. A soaking period of approximately 30 days can be considered a practical soaking endpoint to achieve a relatively stable and reproducible taste profile, supporting process optimization and standardization of garlic-flavored light soy sauce.

## 1 Introduction

In multi-component food systems, flavor development is shaped not only by the intrinsic composition of individual ingredients but also by dynamic interactions between the food matrix and added raw materials. The migration of non-volatile taste-active compounds, such as free amino acids (FAAs) and soluble peptides, is commonly driven by concentration gradients and further modulated by matrix-dependent partitioning and binding, often evolving from rapid release to gradual stabilization (1–4). However, systematic investigations into the migration behavior of non-volatile taste-active compounds in composite condiments, particularly under practical processing conditions, remain scarce.

Garlic (*Allium sativum* Linn.) is a traditional seasoning ingredient whose sensory characteristics arise from both volatile aroma compounds and non-volatile taste-active substances. The non-volatile fraction of garlic contains a diverse pool of free amino acids (FAAs); glutamic acid, glutamine, arginine, and aspartic acid are commonly reported as abundant components, and umami-related FAAs (e.g., glutamic acid and aspartic acid) are closely associated with umami perception and overall taste attributes (5). Light soy sauce is a complex fermented condiment, and its flavor formation involves multiple components, including amino acids and Maillard reaction products (6). Previous studies have mainly focused on garlic-related flavor stability (7) and compositional changes during soy sauce fermentation (8), whereas evidence on garlic–soy sauce composite systems during soaking remains limited. In this context, instrumental taste profiling using an electronic tongue coupled with chemometrics has been applied to relate compositional features (including amino-acid-related features) to taste perception in complex food matrices (9). Umami is a key determinant of food palatability, and its perception is closely associated with umami-related amino acids, particularly glutamate (10). Because FAAs are relatively stable and quantifiable, they provide a suitable basis for tracking taste modulation in garlic–soy sauce systems.

Several gaps remain for garlic soaking systems. First, the dynamic and stage-dependent migration of garlic-derived non-volatile taste-active compounds (especially FAAs) into the soy sauce matrix has rarely been quantified in a time-resolved manner. Second, the mechanistic contribution of non-volatile components to taste formation during soaking is still insufficiently characterized, including whether observed FAA changes are mainly governed by solid–liquid mass transfer or jointly shaped by matrix binding/release and in-system transformation. Third, few studies integrate electronic tongue profiling and targeted FAA quantification with screening tools such as taste activity values (TAVs) and orthogonal partial least squares–discriminant analysis (OPLS-DA) with variable importance in projection (VIP) values to identify key taste-active compounds and to determine a practical soaking endpoint.

Accordingly, we integrated electronic tongue profiling (global taste patterns) with targeted FAA quantification (compound-level evidence), and further applied TAV and OPLS-DA/VIP screening to link migration-driven compositional changes to taste evolution and to identify key contributors across soaking stages.

To address these gaps, we propose a testable, stage-dependent hypothesis for FAA dynamics in the garlic–soy sauce system. The early phase is dominated by solid–liquid mass transfer (leaching/diffusion), leading to rapid changes in FAA profiles (and their TAVs) together with the overall taste profile; this is followed by a slower phase in which reduced concentration gradients and matrix-dependent partitioning/binding drive gradual stabilization (1–4). Based on this hypothesis, the present study establishes an integrated analytical framework to monitor the migration of taste-active FAAs from garlic into light soy sauce, relate these changes to taste profile evolution, and apply complementary statistical screening (TAV and OPLS-DA/VIP) to identify core taste-active compounds across soaking stages. The aim of this work was to elucidate the time-dependent migration behavior of garlic-derived FAAs and their contributions to taste formation, thereby providing a practical time reference for process optimization and standardized production of garlic-flavored light soy sauce.

## 2 Materials and methods

### 2.1 Materials and instruments

Garlic (*Allium sativum*; Hanyuan, Sichuan, China) and light soy sauce (550 mL; premium-grade light soy sauce produced by a local enterprise in Sichuan, China) were used in this study. Plate Count Agar, Potato Dextrose Agar (Beijing Luqiao Technology Co., Ltd.). Fructose standard (CAS 7660-25-5, purity ≥ 98%; Shanghai Shifeng Biological Technology Co., Ltd., Shanghai, China), caffeine standard (CAS 58–08-2, purity ≥ 99%; Shanghai Shifeng Biological Technology Co., Ltd., Shanghai, China), mixed amino acid standard solution, and ninhydrin reagent (Sykam GmbH, Eresing, Germany) were employed. Concentrated hydrochloric acid, sulfosalicylic acid, and sodium hydroxide (analytical grade; Sinopharm Group, China) were also used. Other commonly used laboratory reagents were of analytical grade.

### 2.2 Sample preparation

The soaking conditions were fixed to ensure experimental comparability and were not intended as a universal manufacturing standard. To establish a controlled and reproducible soaking model, peeled garlic cloves were sliced to approximately 2 mm thickness using a sterile knife. The slices were mixed with commercial premium-grade light soy sauce (salt content 16%, pH = 5.1) at a mass ratio of 1:5 (garlic:soy sauce), and gently stirred to ensure full immersion and uniform mixing. The mixture was immediately aliquoted into sterilized amber glass bottles (250 mL capacity). Each bottle was filled to 150 mL (leaving headspace) and then sealed. All bottles were stored in the dark in a biochemical incubator maintained at 25 °C, with occasional gentle shaking to ensure homogeneity. Five sampling time points were established: day 0 (A, blank control, without garlic), day 10 (B), day 20 (C), day 30 (D), and day 40 (E); Group A contained pure light soy sauce without garlic. For each sampling group (A–E), eight independently prepared bottles (150 mL per bottle) were set up as replicates. At each designated sampling time, the bottle contents were filtered through sterile gauze to remove garlic slices and visible particulates, and the filtrate (soy sauce phase) was collected for analysis. Based on preliminary electronic tongue and FAA data showing only minor changes between day 30 and day 40, day 40 was included as the final sampling point to verify whether the soaking process approached a plateau within this window. Three bottles were randomly selected for electronic tongue and free amino acid analyses and used for statistical comparisons (*n* = 3 independent preparations); the remaining five bottles were retained as reserve samples and stored at 4 °C for later use.

### 2.3 Microbial analysis

Microbiological analyses were performed according to GB 2717–2018 and GB 4789.15–2016 (People’s Republic of China).

### 2.4 Electronic tongue analysis

Electronic tongue analysis was performed using an Astree electronic tongue (sixth-generation sensor set; Alpha MOS, Toulouse, France).

A 10 mL aliquot of each sample was diluted to 100 mL with ultrapure water and filtered; 80 mL of the filtrate was used for measurement. Beakers were placed on the autosampler, and the measurement time was set to 120 s followed by 120 s of sensor cleaning. Each sample was measured for 30 consecutive cycles, and the response at 120 s was recorded; the last 10 stable cycles with RSD ≤ 5% across all channels were averaged for subsequent analysis.

Taste intensity radar charts were generated using the Alpha MOS (AlphaSoft) taste-intensity module. Sweetness and bitterness were calibrated using fructose and caffeine standards, respectively, with at least five concentration levels (including blank), and sample intensities were reported as fructose- and caffeine-equivalent values (11).

### 2.5 Amino acid analysis

Free amino acids were determined using a fully automatic amino acid analyzer (S-433D; Sykam GmbH, Eresing, Germany) with ninhydrin derivatization.

A 1.00 mL aliquot of soy sauce filtrate from each time point was transferred into a 50 mL volumetric flask and mixed with 40 mL of 0.01 M HCl. The mixture was vortexed for 5 min, sonicated for 20 min, and then brought to volume with ultrapure water. After standing in the dark for 2 h, a 5 mL aliquot was transferred to a 15 mL centrifuge tube and centrifuged at 4 °C and 10,000 rpm for 4 min. Subsequently, 1.00 mL of the supernatant was mixed with 9.00 mL of 1% (w/v) sulfosalicylic acid, vortexed for 10 min, and allowed to stand in the dark for 1 h, followed by a second centrifugation at 4 °C and 10,000 rpm for 5 min. Finally, the supernatant was filtered through a 0.22 μm aqueous membrane and used for free amino acid determination. This pretreatment was written based on published soy-sauce FAA workflows (acidified dilution/extraction–SSA deproteinization–0.22 μm filtration), with matrix- and dilution-specific procedural details adapted to the present study (12, 13).

An LCA K07/Li chromatographic column (150 mm × 4.6 mm) was used, with an injection volume of 50 μL. The detection wavelengths were set at 570 nm and 440 nm. The flow rate of the ninhydrin reagent mobile phase was maintained at 0.25 mL/min, and the total analysis time was 108 min.

### 2.6 Statistical analysis

To describe the apparent migration behavior, the net change of each FAA in the soy sauce phase was evaluated relative to the blank control (day 0). The relative change (%) was calculated as [(c*_t_* − c*_0_*)/c*_0_*] × 100, where c*_t_* is the FAA concentration at soaking time *t* (10, 20, 30, or 40 d) and c*_0_* is the concentration in the blank soy sauce (day 0, without garlic). Taste activity values (TAVs) were calculated to evaluate the contribution of individual amino acids to taste, using TAV*_i_* = C*_i_*/T*_i_*, where C*i* is the measured concentration of amino acid *i* in the sample and T*_i_* is the corresponding taste threshold reported in the literature. Amino acids with TAV > 1 were considered taste-active, and the total TAV was calculated as the sum of TAVs of taste-active amino acids (14). Based on the raw data from the electronic tongue sensors, taste intensity was analyzed using the taste intensity module of Alpha MOS 17.0 software, and Mahalanobis distances between samples were calculated with its similarity module. Taste intensity radar charts were plotted using Origin 2021. Dimensionality reduction on the sensor response values was performed using the PCA (principal component analysis) plug-in in Origin 2021, from which PCA plots were generated. All data are expressed as mean ± standard deviation (SD). Significant differences among migration stages were assessed by one-way analysis of variance (ANOVA) followed by *post hoc* Tukey’s HSD test (*α* = 0.05), conducted in SPSS 26.0 (IBM Corporation, United States). To discriminate samples based on their free amino acid profiles, OPLS-DA was performed in SIMCA 14.1. Free amino acid contents were set as X variables, and the five groups (blank, 10, 20, 30, and 40 days) were used as Y-class labels. Automatic modeling was performed using the OPLS-DA (Orthogonal Partial Least Squares Discriminant Analysis) module. The resulting model was evaluated using score plots, clustering analysis, and a permutation test (200 iterations). VIP (Variable Importance in Projection, VIP) analysis was then applied to identify key discriminative amino acids. Finally, Pearson correlation analysis between electronic tongue taste intensities and amino-acid TAV values was performed using Origin 2021.

## 3 Results and discussion

### 3.1 The total plate count, changes in mold and yeast during the migration of garlic in soy sauce

**Figure 1:**
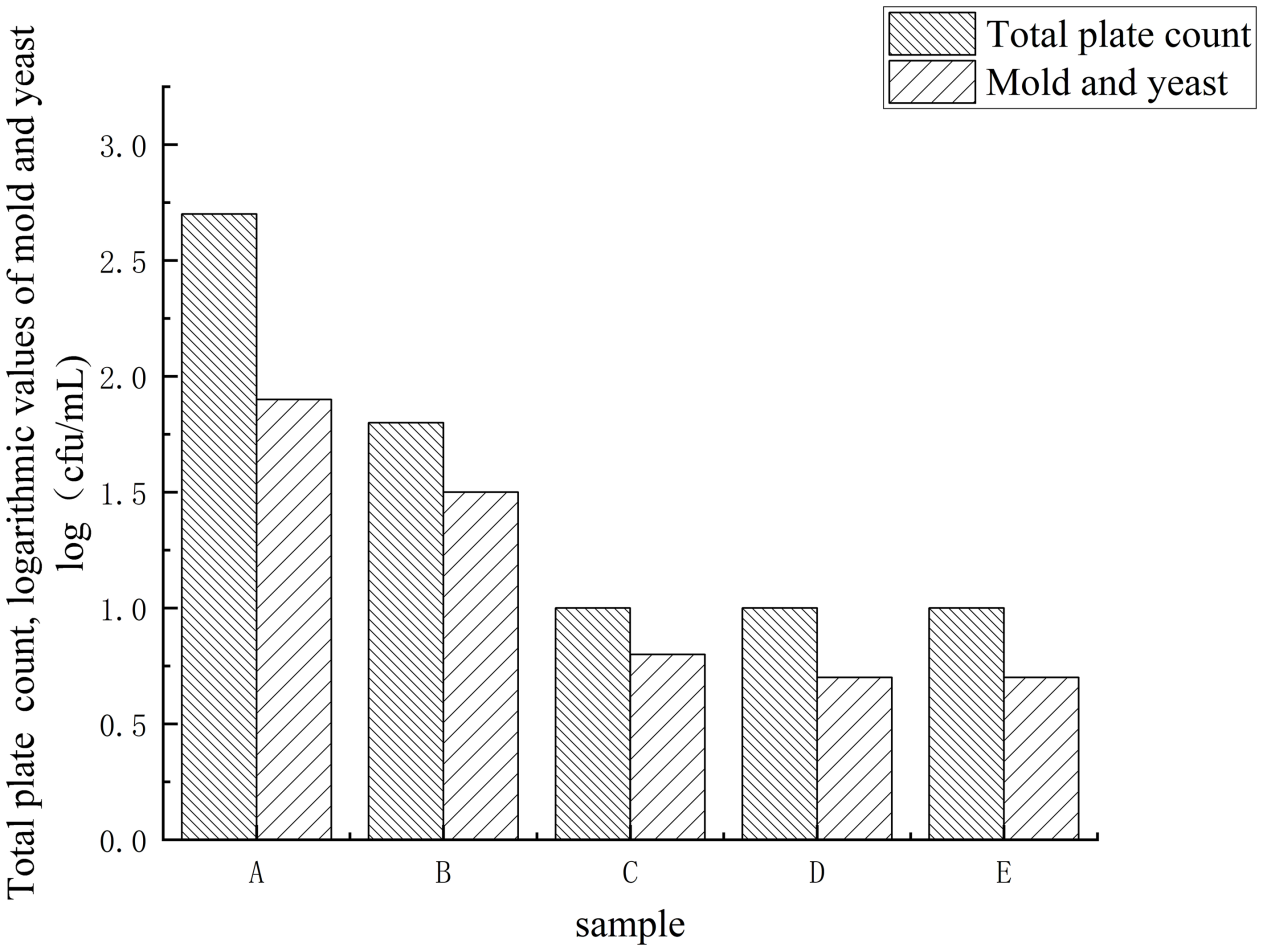
The total plate count, changes in mold and yeast during the migration of garlic in soy sauce. Sample codes day 0 (A, blank control, without garlic), day 10 (B), day 20 (C), day 30 (D), and day 40 (E). Soaking conditions: 25 °C, dark storage.

To exclude spoilage-related taste deviations, total viable counts (TVC) and yeast/mold counts were tracked throughout the soaking process (Figure 1). Compared with the blank soy sauce (A), the garlic-soaked groups (B–E) exhibited lower microbial loads, and counts either decreased or remained at a low, stable level over time. Importantly, no upward trend was observed at later stages (30–40 d), indicating that the system did not enter a microbial growth phase under the present storage conditions.

This suppression is consistent with the combined hurdles of high salt (16%), dark storage, and garlic-derived antimicrobial constituents (e.g., allicin and related sulfur compounds) (7), together with the low-water-activity and inhibitory matrix of soy sauce. Therefore, the time-dependent taste shifts detected by the electronic tongue are unlikely to be driven by microbial spoilage or secondary fermentation, and are more plausibly attributed to physicochemical migration and accumulation of garlic-derived soluble components in the soy sauce phase, which is further examined below using FAA profiling and multivariate analysis.

### 3.2 Changes in taste attributes during soaking (electronic-tongue)

**Figure 2:**
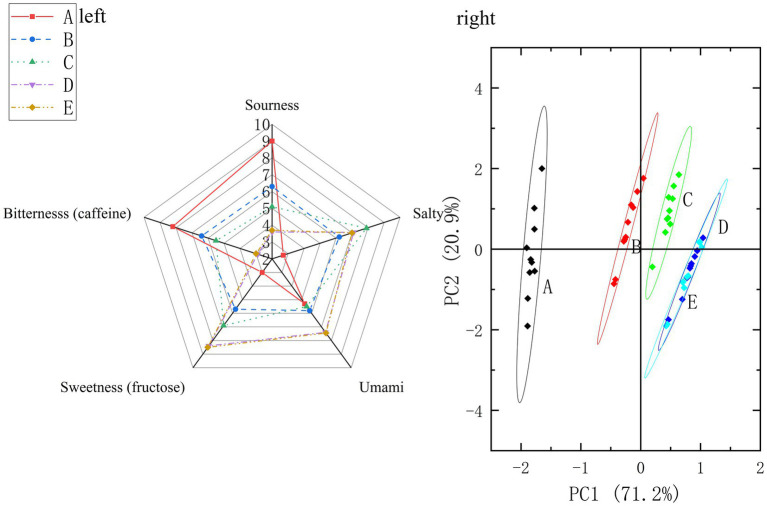
Electronic tongue taste intensity radar chart and PCA during garlic soaking in light soy sauce. (Left) Radar chart of taste intensity indices (sourness, sweetness, umami, saltiness, and bitterness; arbitrary units) generated by the Alpha MOS taste-intensity module. (Right) PCA score plot based on raw sensor responses. Sweetness and bitterness indices were calibrated using fructose and caffeine standard solutions, respectively (intensity-equivalent scaling; not chemical quantification). Sample codes day 0 (A, blank control, without garlic), day 10 (B), day 20 (C), day 30 (D), and day 40 (E). Soaking conditions: 25 °C, dark storage. Radar values represent mean taste-intensity indices predicted by the Alpha MOS module (arbitrary units; *n* = 3 independent sample preparations). Statistical comparisons among groups are summarized in Supplementary Table S1 (one-way ANOVA with Tukey’s HSD test, *p* < 0.05).

The taste profile evolution during garlic soaking was monitored using an electronic tongue. Radar charts provide a direct comparison of differences among samples across the targeted taste dimensions. Statistical analysis of the electronic tongue data (one-way ANOVA with Tukey’s HSD test, *α* = 0.05) revealed significant changes in all five taste indices over the migration period (for detailed mean ± SD values and significance groupings, see Supplementary Table S1). Figure 2 (left) shows the taste intensity radar charts of garlic-derived taste compounds in light soy sauce at different migration times (blank, day 10, day 20, day 30, and day 40). The results indicated that sourness and bitterness intensities exhibited a decreasing trend of A (blank) > B > C > D ≈ E, suggesting that the reduction in sourness (e.g., organic acids) and bitterness (e.g., polyphenolic compounds) in soy sauce with prolonged migration time may be associated with the neutralization or adsorption effects of alkaline components in garlic (e.g., basic amino acids) (15). Sweetness intensity increased with migration time (A < B < C < D ≈ E), indicating that sweet tasting substances in garlic (e.g., fructose or sweet amino acids) gradually migrated into the soy sauce, potentially synergizing with Maillard reaction products in the soy sauce matrix to enhance sweetness perception (16). Umami intensity remained comparable among samples A–C (*p* > 0.05; Supplementary Table S1) and increased significantly in samples D and E (*p* < 0.05), indicating that extended soaking promoted the accumulation of umami-active components, while minor mid-stage fluctuations may be influenced by matrix interactions (17). To further explain why the umami increase can persist over an extended soaking period, the subsequent increase in umami intensity in samples D and E likely reflects sustained diffusion-limited transfer of umami-active FAAs from garlic tissues, together with matrix-dependent partitioning/binding and gradual re-equilibration in the soy sauce phase, which allows continued net accumulation of key umami contributors (notably glutamic acid) at later stages. Saltiness increased sharply after soaking and reached the highest mean value at day 20; values at days 30 and 40 remained high with no significant difference from day 20 (Supplementary Table S1), indicating that saltiness perception approached a plateau in the later stage under the present conditions. Here, the electronic-tongue “saltiness” index is interpreted as an apparent sensor-response intensity influenced by ionic strength and matrix interactions rather than a direct measurement of NaCl content; therefore, the observed peak-and-plateau pattern is discussed cautiously as a combined effect of solute/ion release from garlic tissues and gradual re-equilibration in the soy sauce matrix. While the radar chart delineates the evolution of individual taste attributes, a multivariate approach is required to visualize the holistic taste trajectory and inter-sample relationships.

To this end, principal component analysis (PCA) was performed on the electronic-tongue data (Figure 2, right). The cumulative contribution of PC1 and PC2 was 92.1%, indicating that the dimensionality reduction process effectively extracted and retained a large amount of key information, demonstrating good reduction performance (16–18). Within the 95% confidence ellipse, samples D and E overlapped, confirming their high similarity and marking the stable phase of the migration process. In addition, samples B and C showed relatively small differences, while all samples containing garlic (B, C, D, E) were clearly separated from the blank control A. Taste-intensity differences and statistical comparisons are provided in Supplementary Table S1. From the distribution trend, the five samples were arranged along the X-axis from left to right in the order of migration time, indicating that garlic-derived taste compounds gradually migrated into the light soy sauce, leading to varying degrees of garlic taste characteristics over different migration periods. This chronological distribution along PC1 underscores the dominant role of soaking time in driving overall taste differentiation.

Despite the clear time-dependent trend observed in PCA, the radar chart revealed that the migration dynamics of specific tastes were not uniform. The electronic-tongue results indicated that the migration patterns of certain taste compounds exhibited a non-linear trend. Among these attributes, umami and saltiness showed non-linear fluctuations. This may reflect multiple interacting factors, including polyphenol binding, bitterness antagonism, and polysaccharide adsorption (17). However, PCA indicated a clear time-ordered shift of the overall taste profile along the soaking gradient, suggesting that soaking time was a major driver of global taste differentiation despite local non-linear fluctuations. In addition, day 30 and day 40 samples were statistically indistinguishable for all five taste indices (*p* > 0.05; Supplementary Table S1), indicating limited additional change in the electronic tongue taste profile between 30 and 40 days under the present conditions. Collectively, these results suggest that about 30 days may be a practical endpoint at which the electronic tongue taste profile shows limited additional change under the present conditions.

This conclusion is further corroborated by Mahalanobis distance analysis (Supplementary Table S2). The data showed that the Mahalanobis distance between D and E was the smallest (0.28), followed by that between B and C (1.84), whereas the distances between A (blank) and all other samples were greater than 3.8, indicating that A (blank) differed significantly from the other samples. This result was consistent with the conclusion drawn from the principal component analysis.

The electronic tongue proved to be an effective tool for taste profiling in this complex system, providing objective, quantitative data that maps overall trends and identifies stabilization points. While it cannot fully replace human sensory evaluation for capturing the full multidimensional experience, its data serve as a valuable basis for guiding subsequent targeted chemical analysis. However, a key limitation of the electronic-tongue is its inability to identify the specific compounds responsible for the observed sensory shifts. Accordingly, combining instrumental profiling with targeted chemical analysis has been commonly adopted to interpret flavor changes in complex food systems (19, 20). Therefore, to uncover the key non-volatile taste-active substances underlying these profile changes, targeted free amino acid analysis was conducted.

### 3.3 Changes in free amino acids during the soaking process

#### 3.3.1 Changes in free amino acid composition

Based on the electronic-tongue results, targeted free amino acid (FAA) profiling was performed to identify non-volatile contributors underlying taste evolution. Here, “migration” refers to the apparent net accumulation of FAAs in the soy sauce liquid phase during garlic soaking, quantified by time-resolved FAA profiling relative to the blank control. As summarized in Table 1, 16 FAAs were detected (seven essential amino acids for adults plus histidine; tryptophan was not detected). Total FAA content increased from 31.66 mg/g in the blank (A) to 35.39 mg/g at 10 d (B) and reached a maximum at 20 d (C, 37.80 mg/g), followed by a slight decline and stabilization at 30–40 d (D–E, 36.57–36.49 mg/g). This increase-then-plateau pattern suggests a rapid leaching phase during 0–20 d followed by a slower equilibration stage.

**Table 1:** Free amino acid concentrations in light soy sauce during garlic soaking.

No	Compound	A (mg/g)	B (mg/g)	C (mg/g)	D (mg/g)	E (mg/g)	Taste characteristics*
1	Aspartic acid	3.28 ± 0.41^b^	4.36 ± 0.26^a^	4.38 ± 0.30^a^	3.87 ± 0.35^ab^	3.90 ± 0.27^ab^	umami
2	Glutamic acid	3.67 ± 0.28^b^	3.71 ± 0.18^b^	5.42 ± 0.46^a^	5.98 ± 0.39^a^	5.95 ± 0.27^a^	umami
3	Glycine	1.65 ± 0.14^a^	1.30 ± 0.01^b^	1.32 ± 0.12^b^	1.29 ± 0.10^b^	1.30 ± 0.05^b^	umami
4	Alanine	2.10 ± 0.10^a^	2.35 ± 0.20^a^	2.31 ± 0.07^a^	2.22 ± 0.08^a^	2.21 ± 0.17^a^	umami
5	Lysine	1.38 ± 0.10^b^	2.08 ± 0.12^a^	1.94 ± 0.21^a^	2.23 ± 0.05^a^	2.21 ± 0.21^a^	umami
6	Threonine	1.86 ± 0.12^b^	1.78 ± 0.07^b^	2.12 ± 0.15^a^	1.82 ± 0.11^b^	1.81 ± 0.05^b^	sweetness
7	Serine	2.25 ± 0.03^b^	2.67 ± 0.24^a^	2.74 ± 0.14^a^	2.57 ± 0.22^ab^	2.56 ± 0.02^ab^	sweetness
8	Histidine	0.62 ± 0.05^b^	0.83 ± 0.06^a^	0.84 ± 0.06^a^	0.88 ± 0.05^a^	0.85 ± 0.04^a^	sweetness
9	Valine	5.12 ± 0.10^a^	5.34 ± 0.42^a^	5.41 ± 0.36^a^	5.11 ± 0.28^a^	5.11 ± 0.03^a^	bitterness
10	Methionine	0.72 ± 0.06^a^	0.64 ± 0.04^ab^	0.71 ± 0.05^a^	0.62 ± 0.03^ab^	0.61 ± 0.04^ab^	bitterness
11	Isoleucine	2.47 ± 0.15^a^	2.43 ± 0.16^a^	2.55 ± 0.12^a^	2.32 ± 0.20^a^	2.34 ± 0.17^a^	bitterness
12	Leucine	2.64 ± 0.14^b^	3.07 ± 0.11^a^	3.31 ± 0.18^a^	3.05 ± 0.13^a^	3.06 ± 0.30^a^	bitterness
13	Tyrosine	0.50 ± 0.03^a^	0.50 ± 0.02^a^	0.51 ± 0.01^a^	0.40 ± 0.04^b^	0.42 ± 0.03^b^	bitterness
14	Phenylalanine	1.98 ± 0.16^b^	2.25 ± 0.12^ab^	2.32 ± 0.20^a^	2.18 ± 0.10^ab^	2.16 ± 0.20^ab^	bitterness
15	Arginine	1.34 ± 0.01^b^	2.01 ± 0.11^a^	1.86 ± 0.15^a^	1.97 ± 0.18^a^	1.94 ± 0.09^a^	bitterness
16	Cystine	0.08 ± 0.00^a^	0.07 ± 0.00^a^	0.06 ± 0.00^a^	0.06 ± 0.00^a^	0.06 ± 0.00^a^	ND
Total amino acid content		31.66 ± 0.92^c^	35.39 ± 0.33^b^	37.80 ± 0.46^a^	36.57 ± 0.68^ab^	36.49 ± 0.75^ab^	
Total essential amino acid content†		16.17 ± 0.30^c^	17.59 ± 0.31^ab^	18.36 ± 0.55^a^	17.33 ± 0.41^b^	17.30 ± 0.17^b^	
Total umami amino acid content		12.08 ± 0.82^c^	13.80 ± 0.27^b^	15.37 ± 0.03^a^	15.59 ± 0.63^a^	15.57 ± 0.51^a^	
Total sweet amino acid content		4.73 ± 0.10^c^	5.28 ± 0.26^b^	5.70 ± 0.09^a^	5.27 ± 0.14^b^	5.22 ± 0.06^b^	
Total bitter amino acid content‡		14.77 ± 0.34^b^	16.24 ± 0.48^a^	16.67 ± 0.56^a^	15.65 ± 0.35^ab^	15.64 ± 0.22^ab^	

Sample codes: day 0 (A, blank control, without garlic), day 10 (B), day 20 (C), day 30 (D), and day 40 (E). Soaking conditions: 25 °C, dark storage. Data are presented as mean ± standard deviation (SD) (*n* = 3). Significant differences among soaking times were analyzed by one-way ANOVA followed by Tukey’s HSD test (*α* = 0.05). Different lowercase superscript letters within the same row indicate significant differences among soaking times (*p* < 0.05), whereas the same letters indicate no significant difference (*p* > 0.05). *Taste characteristics were assigned according to Kawai et al. (1). ND indicates no distinct taste category. †Total essential amino acid content was calculated as the sum of Thr, Val, Met, Ile, Leu, Phe, and Lys (consistent with the original table definition). ‡Total bitter amino acid content was calculated as the sum of Val, Met, Ile, Leu, Tyr, Phe, and Arg (Cystine was not included and is reported separately as ND).

Grouping FAAs by taste characteristics further illustrates stage-specific shifts. Umami-type FAAs increased steadily (12.08 → 13.80 → 15.37 → 15.59 → 15.57 mg/g), whereas sweet and bitter FAA pools peaked at 20 d and then slightly decreased (sweet: 4.73 → 5.28 → 5.70 → 5.27 → 5.22 mg/g; bitter: 14.85 → 16.31 → 16.73 → 15.71 → 15.70 mg/g). Essential amino acids showed a similar peak at 20 d (16.17 → 17.59 → 18.36 mg/g) and then decreased to ~17.30 mg/g at 30–40 d. Because microbiological monitoring indicated no progressive microbial growth (Section 3.1), the post-20 d decline is more likely explained by physicochemical factors, such as reduced concentration gradients, matrix-dependent partitioning/adsorption, and slow non-enzymatic reactions in the soy sauce background (16, 17).

Notably, glutamic acid exhibited a distinct trajectory compared with total FAAs. Its concentration increased continuously and peaked at 30 d, rising by 1.01, 47.68, 62.94, and 62.13% in B–E versus the blank. This selective enrichment of glutamate provides a direct compositional basis for the pronounced increase in umami intensity after 30 d (Table 1), even though total FAAs were already maximal at 20 d. The slight (non-significant) dip in electronic-tongue umami from 10 to 20 d may reflect matrix effects rather than insufficient glutamate, including binding of nitrogenous umami-related substances to polyphenols in soy sauce and temporary suppression by bitter amino acids (e.g., valine) through taste–taste antagonism (10, 17). As soaking proceeds and the system approaches a new equilibrium, the persistent accumulation of glutamate together with stabilization of interfering components likely contributes to the observed umami enhancement and overall taste stabilization around 30 d.

#### 3.3.2 TAV analysis of free amino acids

Table 2 presents the TAV analysis results of free amino acids in light soy sauce at different migration times. Previous research (21) has indicated that when TAV > 1, the corresponding amino acid can be considered to make a significant contribution to the overall taste. The data showed that 12 amino acids, including glutamic acid (TAV = 12.23–19.93) and valine (TAV = 12.80–13.53), had TAV values greater than 1, indicating that these amino acids were key contributors to the taste profile of soy sauce. The cumulative TAV values of umami amino acids (aspartic acid, glutamic acid, glycine, alanine, and lysine) increased with migration time, suggesting a progressive enhancement of umami-related taste potential across the soaking period (based on umami-amino-acid TAVs and not accounting for matrix interactions). Overall, the umami-amino-acid TAVs increased and reached their highest level at 30 days.

**Table 2:** Taste activity values (TAV) of free amino acids in light soy sauce during garlic soaking.

Compound	Threshold (mg/g) *	A-TAV	B-TAV	C-TAV	D-TAV	E-TAV
Aspartic acid	1	3.28	4.36	4.38	3.87	3.90
Threonine	2.6	0.72	0.68	0.82	0.7	0.70
Serine	3	0.75	0.89	0.91	0.86	0.85
Glutamic acid	0.3	12.23	12.37	18.07	19.93	19.83
Glycine	1.3	1.27	1	1.02	0.99	1.00
Alanine	0.6	3.5	3.92	3.85	3.7	3.68
Valine	0.4	12.8	13.35	13.53	12.78	12.78
Methionine	0.3	2.4	2.13	2.37	2.07	2.03
Isoleucine	0.9	2.74	2.7	2.83	2.58	2.60
Leucine	1.9	1.39	1.62	1.74	1.61	1.61
Tyrosine	0.91	0.55	0.55	0.56	0.44	0.46
Phenylalanine	0.9	2.2	2.5	2.58	2.42	2.40
Histidine	0.2	3.1	4.15	4.2	4.4	4.25
Lysine	0.5	2.76	4.16	3.88	4.46	4.42
Arginine	0.5	2.68	4.02	3.72	3.94	3.88
Total		52.37	58.4	64.46	64.75	64.39

TAV was calculated as concentration divided by taste threshold (mg/g). TAV > 1 indicates a potentially active contributor to taste. Sample codes day 0 (A, blank control, without garlic), day 10 (B), day 20 (C), day 30 (D), and day 40 (E). Soaking conditions: 25 °C, dark storage. Taste thresholds were referenced from Jin et al. (14). Total TAV was calculated as ΣTAV_i and used as a comparative indicator; it does not imply strict additivity of perceived taste intensity due to possible taste interactions.

In addition, valine, as a bitter amino acid, enhanced the complexity of soy sauce taste through synergistic effects with umami amino acids. In terms of total TAV values, the sample at 30 days reached the highest value (64.75) and then began to decrease. In summary, the overall TAV profile reached its maximum at 30 days and then showed a slight decline, suggesting that the system approached a relatively stable stage around day 30 under the present conditions. It can also be inferred that the 30-day sample had the highest umami-related taste potential and a more complex amino-acid taste contribution profile, as reflected by the umami-amino-acid TAVs and total TAV trends.

Total TAV does not imply a strictly additive prediction of sensory intensity, because synergistic or masking interactions among amino acids and matrix components may occur. Therefore, Total TAV is used as a comparative/trend indicator rather than an absolute predictor of perceived taste intensity.

This explains why the umami intensity perceived by electronic tongue did not always linearly correlate with total TAV, as observed in sample C (20 days), where antagonistic effects from bitter amino acids (e.g., valine) might have temporarily suppressed umami perception despite high glutamic acid content. Such interactions underscore the need to interpret TAV trends within the context of the complex matrix.

### 3.4 Multivariate statistical analysis based on OPLS-DA

**Figure 3:**
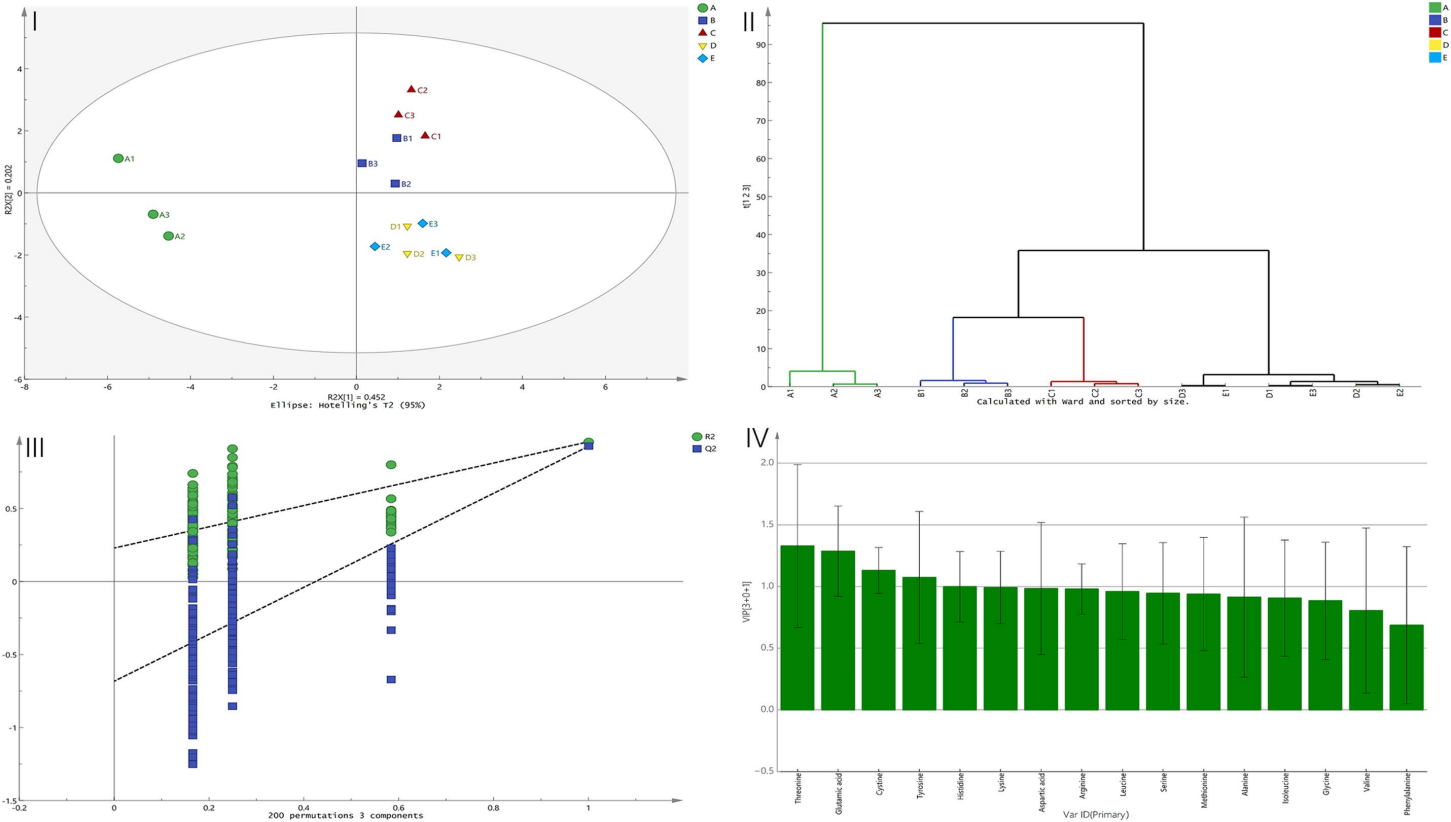
Multivariate analysis based on free amino acid profiles during garlic soaking in light soy sauce. (I) OPLS-DA score plot (SIMCA 14.1; *R*^2^X = 0.738, *R*^2^Y = 0.669, *Q*^2^ = 0.504; 95% confidence interval). (II) Hierarchical clustering analysis (HCA). (III) Permutation test (*n* = 200; R^2^ intercept = 0.229; Q^2^ intercept = −0.683). (IV) VIP analysis (VIP > 1). Sample codes day 0 (A, blank control, without garlic), day 10 (B), day 20 (C), day 30 (D), and day 40 (E). Soaking conditions: 25 °C, dark storage; the input matrix was the free amino acid concentrations (same dataset as Table 1). Amino acids with VIP > 1 include threonine, glutamic acid, cystine, and tyrosine.

Orthogonal partial least squares discriminant analysis (OPLS-DA) is a supervised multivariate statistical method that can extract intergroup difference information more effectively than principal component analysis (PCA), particularly in research scenarios where group differences are pronounced. The model explained X variance (R²X = 0.738) and Y variance (R²Y = 0.669), with a predictive ability of Q² = 0.504. All values exceeded 0.5, indicating that the automatically generated model had good stability and predictive ability (22). Figure 3 (I) shows the OPLS-DA score plot (95% confidence interval). Sample A differed clearly from samples B–E, whereas samples D and E could not be distinguished.

Figure 3 (II) presents the clustering analysis results of the five samples. The plot shows that samples D and E overlapped and were difficult to distinguish, forming the first cluster; subsequently, samples B and C clustered together. These four samples then clustered together before finally clustering with sample A (blank). This result further confirmed that there was a significant difference between the test samples and sample A (blank) (A = Group 1, other samples = Group 2). This conclusion was consistent with the electronic-tongue analysis results, where both principal component analysis and Mahalanobis distance analysis showed the smallest difference between samples D and E and the largest difference between sample A (blank) and the other samples.

Figure 3 (III) shows the results of the permutation test, in which the dependent variable Y matrix was randomly permuted 200 times. The intercept of the *R*^2^ regression line on the Y-axis was 0.229 (< 0.40), indicating that the model had no risk of overfitting; the intercept of the *Q*^2^ regression line on the Y-axis was −0.683 (< 0), indicating that the predictive ability of the established model was reliable (22).

Figure 3 (IV) presents the results of key taste amino acid analysis using the variable importance in projection (VIP) module in SIMCA 14.1 software. Previous studies (23) have shown that when the VIP value is greater than 1, the corresponding compound can be considered a key factor in differentiating samples. VIP analysis identified threonine, glutamic acid, cystine, and tyrosine as having VIP values greater than 1, indicating that these amino acids were the primary contributors to the taste differences among the samples.

VIP screening (VIP > 1) highlighted threonine, glutamic acid, cystine, and tyrosine as the primary discriminants among soaking stages. Beyond glutamate’s consistent umami contribution, these markers also map onto the non-linear behavior of specific taste dimensions observed by the electronic tongue. For example, threonine (sweet-associated) peaked at 20 d (Table 1), coinciding with the steep early rise in sweetness intensity, whereas tyrosine (bitter-associated) decreased significantly at 30–40 d (Table 1), consistent with the marked reduction in bitterness intensity at later stages (Supplementary Table S). Although cystine occurred at low absolute levels, subtle but systematic stage-dependent variation can still contribute to supervised discrimination when combined with other correlated amino acids in the multivariate model. Together, these results indicate that stage separation is driven not only by changes in total FAAs but also by targeted shifts in a small set of sensory-relevant marker amino acids.

The integrated application of PCA, OPLS-DA, and VIP provided complementary perspectives on taste evolution. PCA offered an unsupervised overview of sample clustering, revealing that the overall taste trajectory followed a time-dependent progression, with samples D (30 days) and E (40 days) converging in the score plot (Figure 2, right). OPLS-DA, as a supervised method, enhanced group discrimination and identified the most influential variables (VIP > 1) that drove separation between soaking stages. Notably, the VIP results aligned with the TAV-based screening, with glutamate being highlighted by both approaches. Overall, the unsupervised (PCA) and supervised (OPLS-DA) models showed consistent clustering patterns. Together with the agreement between statistical (VIP) and sensory-driven (TAV) criteria, these results support our conclusion that ~30 days represents a plateau-like stage in the measured taste profile under the present conditions. This multi-method convergence also validates the use of such an integrated framework for identifying key taste-active compounds in complex seasoning systems.

### 3.5 Comparing TAV and VIP for key amino acids

In identifying taste-active compounds in foods, the taste activity value (TAV) and variable importance in projection (VIP) are two complementary analytical approaches, each with distinct principles and emphases (24, 25). The TAV method evaluates the ratio of a compound’s concentration to its taste threshold. Certain low-concentration compounds can still exert a significant influence on flavor due to their extremely low taste thresholds, making TAV an effective indicator for assessing the actual intensity of taste contribution. In this experiment, the TAV method identified 12 compounds with TAV values greater than 1: aspartic acid, glutamic acid, glycine, alanine, valine, methionine, isoleucine, leucine, phenylalanine, histidine, lysine and arginine. In contrast, the VIP method, based on the OPLS-DA model, screens differential compounds among groups through multivariate statistical analysis. In this study, the VIP method identified four characteristic amino acids: threonine, glutamic acid, cystine, and tyrosine. Notably, both methods identified glutamic acid as a core taste-active component, indicating that it had a high contribution value in both evaluation systems.

This finding confirms that the TAV and VIP methods have complementary strengths in the synergistic screening of taste-active compounds. The TAV method focuses on assessing the actual contribution intensity of taste compounds from the perspective of sensory perception (26), whereas the VIP method identifies characteristic markers with group-discriminating ability based on statistical significance (27). Combining the two approaches enables dual validation that considers both sensory contribution intensity and statistical differentiation, thereby achieving a more comprehensive and accurate screening of core flavor compounds. This approach also compensates for the limitations of single methods, which may overlook low-threshold, high-contribution compounds or high-variability, low-impact components, thus providing methodological assurance for the scientific identification of key flavor substances in complex food systems. This dual-validation framework can be adopted as a standardized quality control tool in condiment manufacturing, reducing variability in flavor outcomes. More importantly, the convergence of TAV and VIP methods on glutamic acid as a core taste-active component provides robust, multi-angle validation of its mechanistic importance. The complementary insights from these methods facilitate a more nuanced understanding of taste formation, accounting for both concentration-threshold relationships and statistical differentiation in the complex matrix.

### 3.6 Correlation analysis between electronic-tongue results and amino acids

**Figure 4:**
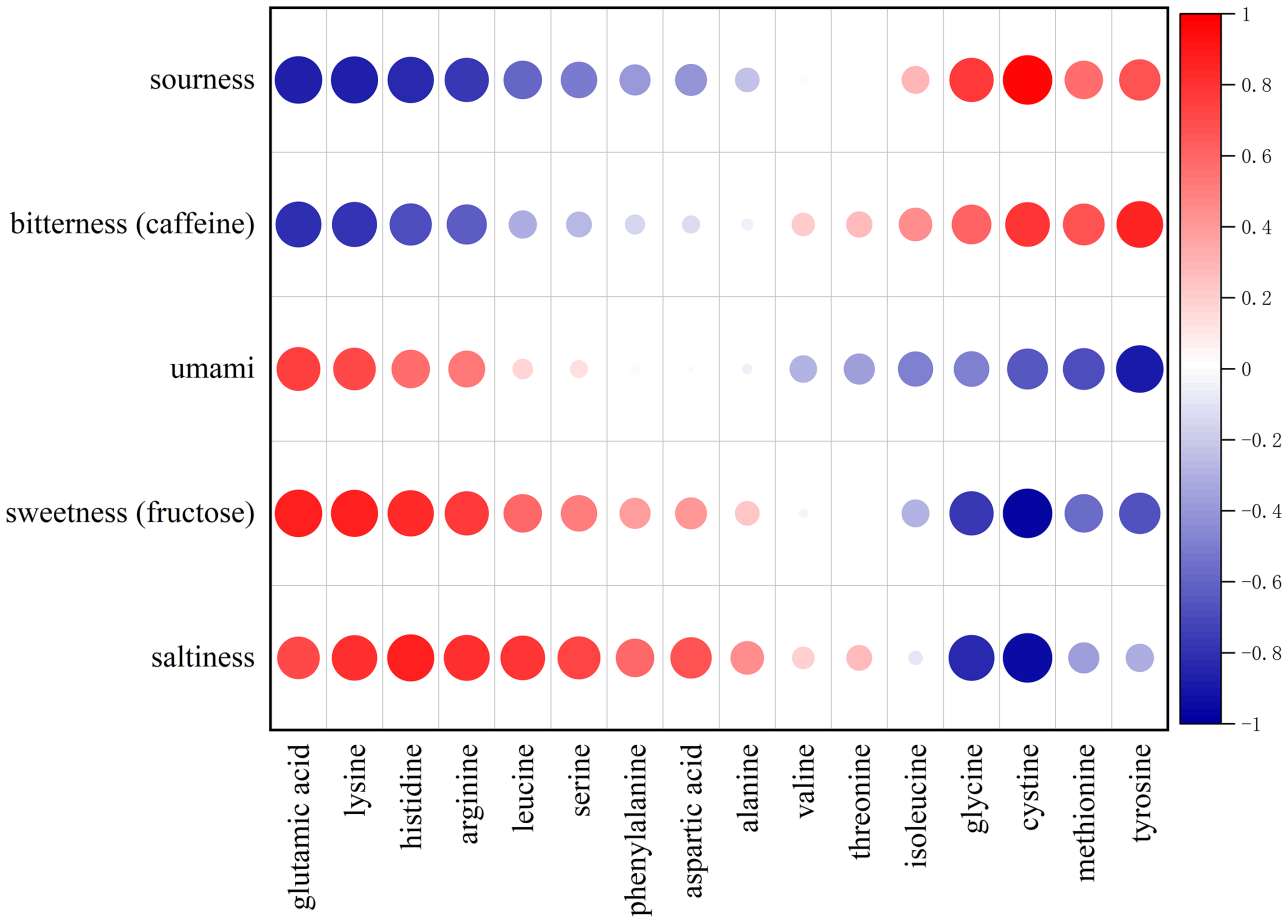
Bubble plot of Pearson correlations between electronic tongue taste intensity indices (sourness, bitterness [caffeine-equivalent], umami, sweetness [fructose-equivalent], and saltiness) and amino-acid taste activity values (TAVs) during garlic soaking in light soy sauce. Notes: Bubble color represents Pearson’s correlation coefficient (*r*), as shown by the color bar (−1 to 1), where red indicates positive and blue indicates negative correlations. Bubble size is proportional to the absolute correlation magnitude (|*r*|); larger bubbles indicate stronger associations. TAVs were calculated as concentration divided by taste threshold (mg/g). Correlations were computed using paired data across migration stages (A–E), where TAV values at each stage (A-TAV…E-TAV) were matched with the electronic tongue indices measured at the same stage.

Figure 4 presents the results of the Pearson correlation analysis (*p* < 0.05) between the taste intensities measured by the electronic tongue and the TAV values of amino acids. The results showed that umami, sweetness, and saltiness were positively correlated to varying degrees with most amino acids, particularly glutamic acid, lysine, histidine, arginine, leucine, serine, phenylalanine, aspartic acid, alanine, valine, and threonine, some of which showed significant correlations. In contrast, tyrosine, methionine, cystine, glycine, and isoleucine were negatively correlated with umami and sweetness, possibly due to their intrinsic bitterness or antagonistic effects on umami perception.

For specific taste attributes, glutamic acid and lysine, as typical umami amino acids, showed a significant positive correlation with umami intensity. In contrast, glycine and alanine, although chemically classified as umami amino acids, showed a negative correlation with umami, suggesting that their sensory performance may be influenced by low concentrations or interactions with other bitter compounds (1).

In terms of sweet amino acids, histidine exhibited the most significant correlation, whereas serine and threonine, despite being classified as sweet amino acids, showed weaker contributions. Saltiness displayed moderate positive correlations with multiple amino acids, indicating that its perception mechanism may be relatively complex.

For bitterness, bitter amino acids such as tyrosine, methionine, isoleucine, and valine showed positive correlations with bitterness intensity, with tyrosine and methionine exhibiting significant positive correlations and having a notable impact on taste formation. In contrast, phenylalanine, leucine, and arginine showed a negative correlation trend, suggesting that their bitterness may be masked or neutralized by other components in the multi-compound matrix (28).

Overall, the taste intensities detected by the electronic tongue showed good correlations with the TAV values, suggesting the effectiveness of the electronic tongue in reflecting the actual flavor characteristics of complex food systems. These results also indirectly validated the feasibility of the TAV method in quantitatively evaluating the contribution of flavor compounds, providing a theoretical basis for constructing multiparameter flavor evaluation models in the future. Such models could be integrated into production monitoring systems, enabling real-time adjustment of soaking time to achieve optimal flavor.

The positive correlations between umami intensity and amino acids such as glutamic acid and lysine are consistent with their established roles as primary umami contributors. These amino acids also show synergistic interactions with 5′-ribonucleotides, which can greatly amplify umami perception beyond additive effects (1, 10). From a molecular taste receptor perspective, the prominence of glutamate and lysine can be mechanistically explained. Glutamate, a canonical umami stimulus, activates the T1R1/T1R3 heterodimeric receptor on taste bud cells, eliciting a strong umami sensation (29, 30). Its consistently high TAV (12.23–19.93) throughout the migration period indicates that its concentration far exceeds the human detection threshold, thereby dominating the umami perception in the garlic soy sauce matrix. Moreover, glutamate is known to exhibit synergistic effects with 5′ ribonucleotides, which may further amplify umami intensity in the complex soy sauce background. Conversely, the negative correlations between umami and amino acids like glycine and alanine, despite their classification as umami-type amino acids, may be explained by their relatively low concentrations in the matrix or by competitive suppression from co-existing bitter compounds, which can mask or attenuate umami perception (31).

For bitterness, strong positive correlations with tyrosine and methionine align with their low taste thresholds and high bitterness potency, whereas the negative correlations of leucine, and arginine with bitterness suggest a masking effect by other dominant taste modalities or a balancing effect in the overall flavor matrix (32). Tyrosine, classified as a bitter-tasting amino acid, primarily acts through TAS2R family bitter taste receptors (33, 34). Its high TAV (12.80–13.53) suggests a significant contribution to bitterness. In composite seasoning systems, moderate bitterness can enhance flavor complexity and balance; however, excessive bitter compounds may antagonize umami perception via receptor competition or central masking. The temporary decrease in umami intensity at 20 days may be attributed to bitter–umami interactions. In this stage, valine and other bitter amino acids may momentarily suppress the umami signal. As the system approaches equilibrium (30 days), the continued migration of glutamate and the stabilization of bitter compounds lead to a harmonious taste profile with enhanced umami and well-integrated bitterness. These patterns reflect the complexity of taste–taste interactions in multi-component food systems, where synergistic and antagonistic effects jointly determine the perceived flavor outcome. Collectively, the correlation analysis not only validates the consistency between instrumental taste measurements and chemical indices (TAV), but also elucidates the mechanistic underpinnings of the observed taste evolution. The strong positive correlations of umami with glutamic acid and lysine confirm their direct contribution, while negative correlations involving tyrosine, methionine illustrate how masking or competitive suppression can modulate overall perception. These insights bridge the gap between experimental patterns (e-tongue profiles) and chemical validation (amino acid migration), offering a coherent explanation for the non-linear taste changes observed during soaking.

Although this study focuses on the migration of garlic-derived free amino acids into light soy sauce, similar mass-transfer patterns are observed in other infusion or fermentation systems. Examples include tea (35), wine (36), and fermented sauces (37). For example, in tea infusion, the migration of polyphenols and amino acids typically shows rapid release followed by stabilization, similar to the migration of free amino acids from garlic in soy sauce. In various fermented condiments, amino acids and small flavor molecules also accumulate in a stage-dependent manner, with faster migration at early stages and gradual stabilization over time. Furthermore, chemical reactions during fermentation (e.g., Maillard reaction, esterification) contribute to flavor evolution. Therefore, the findings of this study provide theoretical support for understanding the migration of flavor compounds in various infusion or fermentation systems.

## 4 Conclusion

This study integrated electronic-tongue profiling, targeted free-amino-acid (FAA) quantification, and dual screening (TAV and OPLS-DA/VIP) to track the evolution of taste formation in garlic-soaked light soy sauce over time. Although total FAAs peaked earlier, glutamate showed sustained accumulation and was consistently identified as the most robust taste-active driver, supporting ~30 days as a practical soaking endpoint for achieving a plateau-like, well-balanced taste profile under the tested conditions.

In addressing key research gaps, this work offers three distinct contributions: (1) systematic characterization of the dynamic migration behavior of non-volatile taste-active compounds in a composite seasoning matrix; (2) integration of multi-instrument approaches (electronic tongue and amino acid analyzer) with dual statistical validation (TAV and VIP) to identify and confirm key taste-active compounds; and (3) establishment of a scientifically grounded soaking endpoint to guide industrial standardization and quality control.

Limitations should be acknowledged. The conclusions were derived under a single formulation and storage condition (one soy sauce system, fixed garlic form and ratio, 25 °C, dark) and within a limited time window (0–40 days), without evaluating post-soaking storage stability, batch-to-batch variability, or microbiological safety. In addition, taste interpretation relied on electronic-tongue responses and literature-based taste thresholds, which may deviate in a high-salt soy-sauce matrix. Moreover, only FAAs were quantified; other non-volatile contributors (e.g., peptides, nucleotides, organic acids, sugars, and polyphenols) and their interactions were not characterized.

Future work should therefore validate the proposed endpoint across raw-material and processing variables (temperature, ratio, salt level, different soy-sauce types), assess chemical/sensory stability during storage, incorporate trained-panel sensory evaluation, and expand targeted profiling to other taste-active non-volatile constituents to clarify synergistic/antagonistic mechanisms.

Overall, the methodological framework and the identified soaking endpoint provide both scientific insight and a practical tool for developing consistent, high-quality garlic-flavored soy sauce with strong potential for industrial application.

## Data Availability

The raw data supporting the conclusions of this article will be made available by the authors, without undue reservation.
