# Supplementary material for: Dynamic migration of free amino acids from garlic in light soy sauce and its impact on taste formation
Source: Front Nutr. 2026 Apr 29;13:1768181. doi: 10.3389/fnut.2026.1768181 (PMC13168135; doi:10.3389/fnut.2026.1768181)
Supplement: Supplementary file 1 [file Table_1.DOCX]

Supplementary Material

# Supplementary Data

Table S1

| Index | A | B | C | D | E |
| --- | --- | --- | --- | --- | --- |
| Sourness | 9.00 ± 0.62^a^ | 6.30 ± 0.46^b^ | 5.10 ± 0.87^bc^ | 3.60 ± 0.36^d^ | 3.70 ± 0.10^cd^ |
| Saltiness | 2.70 ± 0.10^c^ | 6.20 ± 0.46^b^ | 7.90 ± 0.46^a^ | 7.10 ± 0.17^ab^ | 7.00 ± 0.35^ab^ |
| Umami | 5.30 ± 0.30^b^ | 5.80 ± 0.00^b^ | 5.50 ± 0.36^b^ | 7.40 ± 0.46^a^ | 7.45 ± 0.26^a^ |
| Sweetness (fructose equivalent) | 3.00 ± 0.44^c^ | 5.70 ± 0.35^b^ | 6.90 ± 0.26^b^ | 8.40 ± 0.70^a^ | 8.50 ± 0.56^a^ |
| Bitterness (caffeine equivalent) | 8.20 ± 0.87^a^ | 6.40 ± 0.44^b^ | 5.50 ± 0.40^b^ | 2.90 ± 0.26^c^ | 3.00 ± 0.20^c^ |

Table S1. Electronic-tongue taste intensity indices of light soy sauce samples during garlic migration. Values are presented as mean ± standard deviation (n = 3 independent sample preparations). Different superscript letters within the same row indicate significant differences among groups (one-way ANOVA with Tukey’s HSD test, p < 0.05). day 0 (A, blank control, without garlic), day 10 (B), day 20 (C), day 30 (D), and day 40 (E). Soaking conditions: 25 °C, dark storage.
